# Pathogenic Strains of *Acanthamoeba* Are Recognized by TLR4 and Initiated Inflammatory Responses in the Cornea

**DOI:** 10.1371/journal.pone.0092375

**Published:** 2014-03-14

**Authors:** Hassan Alizadeh, Trivendra Tripathi, Mahshid Abdi, Ashley Dawn Smith

**Affiliations:** Department of Cell Biology and Immunology, University of North Texas Health Science Center, and North Texas Eye Research Institute, Fort Worth, Texas, United States of America; UC Berkeley, United States of America

## Abstract

Free-living amoebae of the *Acanthamoeba* species are the causative agent of *Acanthamoeba* keratitis (AK), a sight-threatening corneal infection that causes severe pain and a characteristic ring-shaped corneal infiltrate. Innate immune responses play an important role in resistance against AK. The aim of this study is to determine if Toll-like receptors (TLRs) on corneal epithelial cells are activated by *Acanthamoeba*, leading to initiation of inflammatory responses in the cornea. Human corneal epithelial (HCE) cells constitutively expressed TLR1, TLR2, TLR3, TLR4, and TLR9 mRNA, and *A. castellanii* upregulated TLR4 transcription. Expression of TLR1, TLR2, TLR3, and TLR9 was unchanged when HCE cells were exposed to *A. castellanii*. IL-8 mRNA expression was upregulated in HCE cells exposed to *A. castellanii*. *A. castellanii* and lipopolysaccharide (LPS) induced significant IL-8 production by HCE cells as measured by ELISA. The percentage of total cells positive for TLR4 was higher in *A. castellanii* stimulated HCE cells compared to unstimulated HCE cells. *A. castellanii* induced upregulation of IL-8 in TLR4 expressing human embryonic kidney (HEK)-293 cells, but not TLR3 expressing HEK-293 cells. TLR4 neutralizing antibody inhibited *A. castellanii*-induced IL-8 by HCE and HEK-293 cells. Clinical strains but not soil strains of *Acanthamoeba* activated TLR4 expression in Chinese hamster corneas *in vivo* and *in vitro.* Clinical isolates but not soil isolates of *Acanthamoeba* induced significant (*P*< 0.05) CXCL2 production in Chinese hamster corneas 3 and 7 days after infection, which coincided with increased inflammatory cells in the corneas. Results suggest that pathogenic species of *Acanthamoeba* activate TLR4 and induce production of CXCL2 in the Chinese hamster model of AK. TLR4 may be a potential target in the development of novel treatment strategies in *Acanthamoeba* and other microbial infections that activate TLR4 in corneal cells.

## Introduction

Free-living amoebae of the *Acanthamoeba* species are the causative agent of *Acanthamoeba* keratitis (AK), a sight-threatening corneal infection that causes severe pain and a characteristic ring-shaped corneal infiltrate [1]. *Acanthamoeba* species are ubiquitous in nature; however, not all isolates of *Acanthamoeba* can cause disease since it was found that pathogenic strains of *Acanthamoeba* produce corneal infections in Chinese hamsters *in vivo* [2]. Pathogenesis of AK begins with the attachment of the amoebae to the corneal surface via mannose-binding protein (MBP) [3], [4]. A cytolytic mannose-induced protein (MIP-133) is then secreted by the parasite to aid in the degradation of the corneal layers leading to the parasite’s infiltration around the corneal nerves causing radial neuritis and exquisite pain [5]. Infiltration of inflammatory cells such as macrophages and neutrophils are part of the host’s first line of defense and play an integral role in clearance of the pathogen [6], [7]. Elements of both innate and adaptive immunity are involved in resistance to AK. Several studies suggested that the innate immune response plays a critical role in AK [6], [8], [9] and both *Acanthamoeba* and host factors released from infiltrating cells during infection contribute to a rapidly progressing stromal necrosis [2]. Histopathological analysis of AK lesions in both humans and experimental animals reveals a remarkable inflammatory infiltrate comprised predominantly of neutrophils [10]–[12]. *In vitro* studies have shown that rat and Chinese hamsters’ neutrophils can kill *Acanthamoeba* trophozoites [13]–[14]. *In vivo,* neutrophils influence the course of AK. Inhibition of initial neutrophil migration into corneas of Chinese hamsters infected with *A. castellanii* resulted in a profound exacerbation of AK [6]. It has been reported that the most severe stromal necrosis in AK lesions is in areas of heavy neutrophil infiltration [15]. Further, it has been suggested that stromal necrosis in *Acanthamoeba* lesions is mediated by proteases released by the neutrophils rather than parasitic infection [5], [16]. Therefore, a reduction of polymorphonuclear neutrophils (PMNs) recruitment may be beneficial later in the course of the disease.

Recent studies have shown that epithelial cells also actively participate in the host response to bacterial infection [17]. This first line of defense is affected through recognition of pathogens by Toll-like receptors (TLRs) with subsequent expression and secretion of proinflammatory cytokines and chemokines that recruit inflammatory cells in response to bacterial infection [17], [18]. Toll-like receptors have been shown to have a role in pathogen recognition in bacterial, fungal, and viral keratitis [19], [20]. TLRs are pattern recognition receptors (PRRs) that recognize specific pathogen-associated molecular patterns (PAMPs) leading to the activation of an inflammatory signaling cascade producing proinflammatory cytokines and chemokines [17]. It has been shown that TLRs expressed by the cornea are involved in the recognition of the microbial products that cause keratitis [21]. TLR4 signals through two distinct pathways: a) myeloid differentiating factor-88 (MyD88) dependent and b) MyD88 independent [17]. The MyD88 independent pathway does not use MyD88 and instead uses TRIF (the TIR domain-containing adapter induced IFN-β protein) to induce the activation of IFN-β and interferon induced genes. The MyD88 dependent pathway ultimately leads to the activation of p38, JNK, and NF-κB transcription factors which then activate the expression of proinflammatory genes to produce cytokines and chemokines [22]. The chemokines produced are responsible for the recruitment of PMNs critical to the immune response.

TLR4 does not work alone in the signaling cascade to produce cytokines and chemokines [23]. The receptor works in a complex of proteins that allow for the recognition of its known specific ligand, lipopolysaccharide (LPS) [18]. LPS binding protein (LBP), CD14, and MD-2 are all expressed in the eye and are integral components of the TLR4 recognition system [24], [25]. LBP binds to LPS and transfers the PAMPs onto CD14 [26]. MD-2 is a co-receptor that binds to TLR4 and to LPS making it essential for response [27].

In this study, we determined that pathogenic strains of *Acanthamoeba* are recognized by TLR4 on human and Chinese hamster corneal epithelial (HCORN) cells. We have also investigated the role of TLR4 in the Chinese hamster model of AK. The results indicate that TLR4 is upregulated in human and Chinese hamster corneal epithelial cells following *Acanthamoeba* stimulation. *In vitro* and *in vivo* results showed that pathogenic (Clinical), but not non-pathogenic (Soil) strains of *Acanthamoeba* induced TLR4 activation upon stimulation with *Acanthamoeba* trophozoites leading to significant increase in proinflammatory chemokines production. The present study is the first to compare the *in vitro* and *in vivo* activation of TLR4 simultaneously in response to the infection with pathogenic and non-pathogenic strains of *Acanthamoeba.*

## Results

### *Acanthamoeba* Trophozoites Induce Upregulation of TLR4 Gene Expression in the Corneal Epithelial Cells

**Figure 1:**
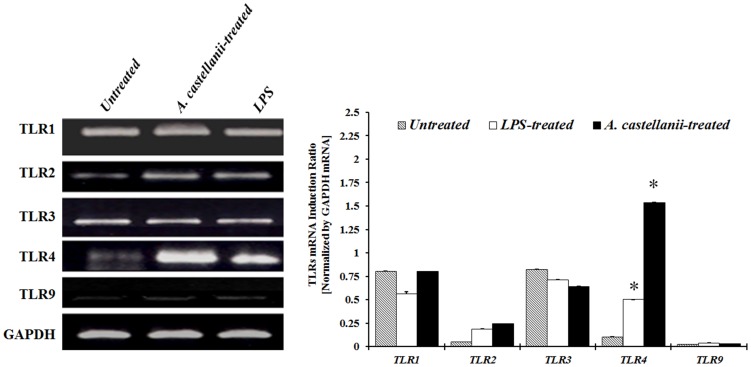
Toll-like receptors gene expression in human corneal epithelial (HCE) cells stimulated with *A. castellanii* trophozoites or LPS. HCE cells were treated with *A. castellanii* trophozoites (1×10^5^ cells/ml) or LPS (10 μg/ml) for 24 hours following which cells were processed for total RNA isolation and RT-PCR. The amount of mRNA expression was quantified by densitometry of bands in comparison to the Glyceraldehyde-3-phosphate dehydrogenase (GAPDH). Densitometry of mRNA bands were quantified by three independent scanned presented as mean±SEM.

To determine if treatment with a pathogenic (Clinical) isolate of *A. castellanii* can activate Toll-like receptors (TLRs) in HCE cells, the corneal epithelial cells were treated with either *A. castellanii* trophozoites, LPS, or left untreated for 24 hours. The expression of TLR1, TLR2, TLR3, TLR4, and TLR9 mRNA was determined by RT-PCR. The results showed an increased expression of TLR4 after treatment (**Figure 1**). All other TLRs tested showed no change in mRNA expression. The results indicate that while several TLRs are expressed constitutively, only TLR4 are involved in *Acanthamoeba* recognition.

### Upregulation of TLR4 and Proinflammatory Cytokine in HCE Cells After *A. castellanii* Treatment

**Figure 2:**
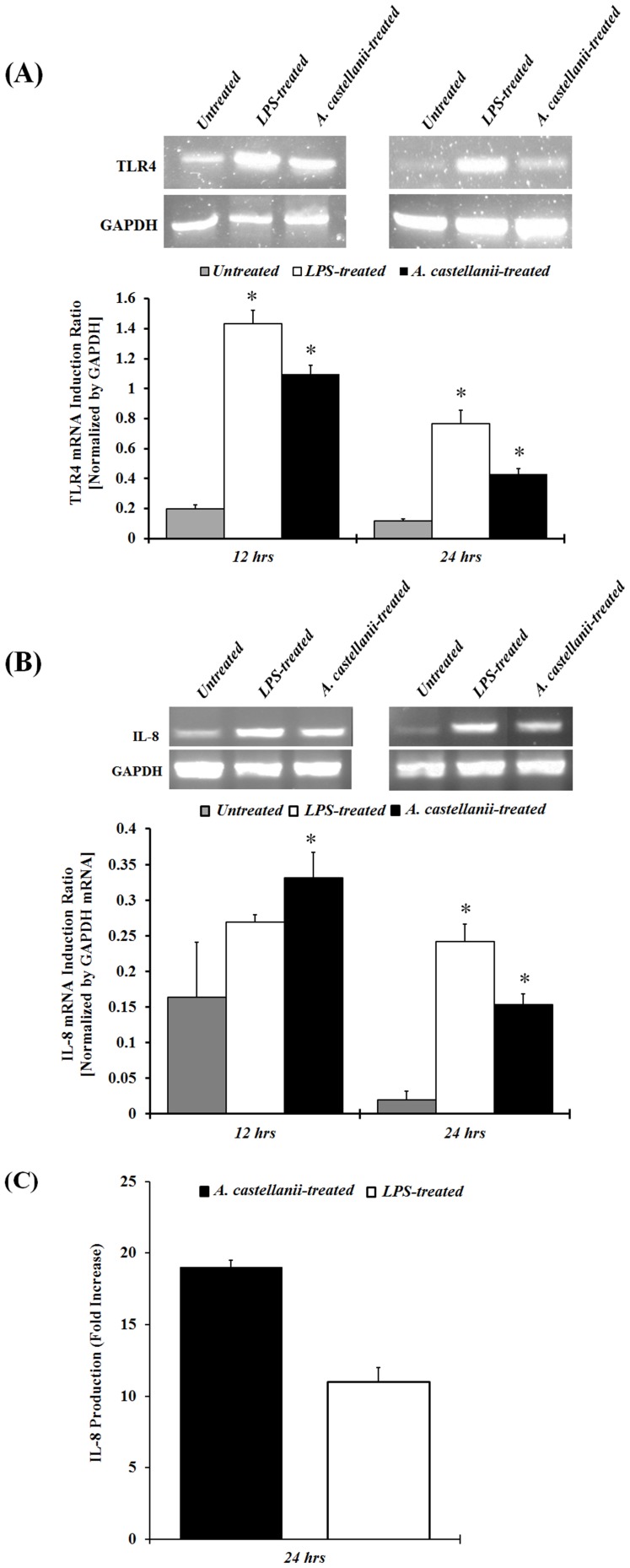
Effect of *A. castellanii* trophozoites on TLR4 and IL-8 mRNA, and IL-8 protein expression in HCE cells. HCE cells were stimulated with *A. castellanii* (1×10^5^ cells/ml) and LPS (10 μg/ml) for 12 and 24 hours, and were then processed for total RNA isolation and RT-PCR analysis for TLR4 and IL-8 mRNA expression. The amount of mRNA expression was quantified by densitometry of bands in comparison to Glyceraldehyde-3-phosphate dehydrogenase (GAPDH). Densitometry of mRNA bands were quantified by three independent scanned presented as mean±SEM **(2A and 2B**). HCE cells were stimulated with *A. castellanii* (1×10^5^ cells/ml) and LPS (10 μg/ml) 24 hours. Supernatants were collected from harvested cells and subjected to IL-8 specific ELISA **(2C**). The data are mean±SEM of three independent experiments. *Asterisk* indicates *P* value < 0.05 by unpaired Student’s *t*-test.

Activation of TLRs is known to induce chemokines gene and protein production by corneal epithelial cells. RT-PCR analysis revealed that *A. castellani* trophozoites induced upregulation of TLR4 and IL-8, 12 and 24 hours after *Acanthamoeba* stimulation *in vitro*. These results suggest that TLR4 and IL-8 gene expression is significantly upregulated at the same time in HCE cells following *Acanthamoeba* stimulation (**Figure 2A and 2B**).

To determine if the increase in chemokine gene expression correlated with an increase in actual IL-8 protein production, HCE cells were cultured with either *A. castellanii* trophozoites or LPS for 24 hours. Cell culture supernatants were collected and analyzed by ELISA. HCE cells stimulated with *A. castellanii* trophozoites produced significantly (*P*< 0.05) more IL-8 than untreated HCE cells (**Figure 2C**). These results indicate that not only is IL-8 gene expression upregulated in HCE cells treated with *A. castellanii* trophozoites, but IL-8 protein production in HCE treated cells comparatively higher than untreated control HCE cells.

### Immunolocalization of TLR4 in HCE Cells

**Figure 3:**
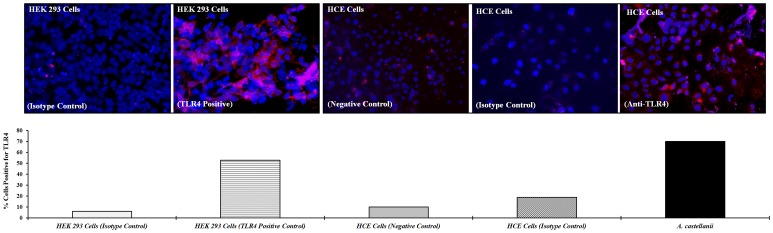
Immunofluorescence antibody staining of TLR4 in HCE cells and TLR4 transfected HEK-293 cells. HCE or HEK-293 cells were grown to confluence on 4 well chamber slides. HCE cells were stimulated with *A. castellanii* trophozoites (1×10^5^ cells/ml) for 24 hours; HCE control cells and HEK-293 (positive control) cells were left untreated for 24 hours at 37°C. After the incubation period, cells were fixed with 4% paraformaldehyde, and then incubated with either PE anti-human TLR4 or PE Mouse IgG2α isotype control for 1 hour. To visualize the nuclei sections were counterstained for one minutes in 150 ng 4,6-diamidino-2-phenylindole, dilactate (DAPI). Three slides in each group were viewed using fluorescence microscopy. Images were captured with an Olympus AX70 Fluorescence Microscope. The results were expressed as percent of cells positive for TLR4 by counting the number of TLR4 positive cells divided by the number of live cells × 100. Cells that stain with DAPI were counted as live cells.

Immunocytochemistry was used to establish the distribution of TLR4 on HCE cell surfaces with and without treatment with *A. castellanii*. TLR4 expressing HEK-293 used as positive control. In control (unstimulated) HCE cells, TLR4 was expressed on the cell membrane with low intensity staining. HCE cells stimulated with *A. castellanii* for 24 hours showed more TLR4 staining cells than unstimulated HCE cells. The percentage of total cells positive for TLR4 was significantly higher (70% *vs.* 10%) in *A. castellanii* stimulated cells compared to HCE control cells. A high intensity staining of TLR4 was demonstrated in HCE and HEK-293 cells (positive control) stimulated with *A. castellanii* (**Figure 3**).

### Activation of TLR4 in HEK-293 Cells by *Acanthamoeba* Increases IL-8 Expression

**Figure 4:**
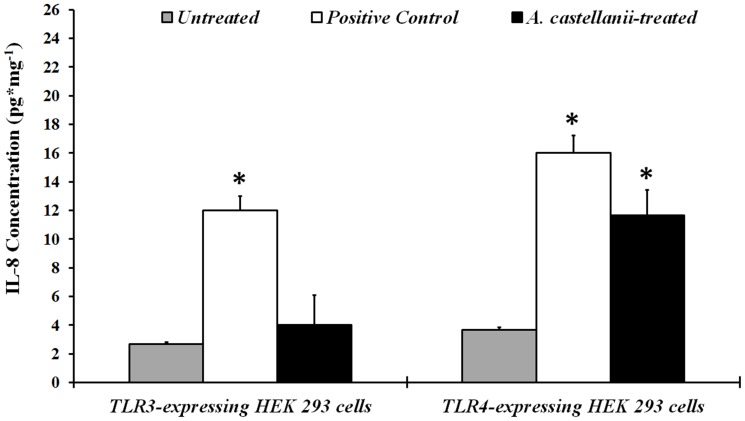
Effect of *A. castellanii* trophozoites on secretion of IL-8 by HEK-293 cells. HEK-293 cells expressing only TLR3 or TLR4 were cultured with *A. castellanii* trophozoites (1×10^5^ cells/ml). As a positive control, HEK-293 cells were activated with 125 ng/ml poly (I:C) (A specific ligand for TLR3) and 100 ng/ml ultrapure LPS (A specific ligand for TLR4/MD2) for 24 hours. Supernatants were collected from harvested cells and subjected to IL-8 specific ELISA. The data are mean±SEM of three independent experiments. *Asterisk* indicates *P* value < 0.05 by unpaired Student’s *t*-test.

To further confirm that TLR4 are involved in the recognition of *Acanthamoeba* and mediate signaling responses to *A. castellanii* trophozoites, TLR3 and TLR4 transfected HEK-293 cells were exposed to *A. castellanii in vitro* for 24 hours. Cells cultured without the trophozoites served as a control. As a positive control, transfected HEK-293 cells were activated with 125 ng/ml poly (I:C) (A specific ligand for TLR3), and 100 ng/ml ultrapure LPS (A specific ligand for TLR4/MD2) for 24 hours as described previously [28]. HEK-293 cells were collected from each well and analyzed for IL-8 mRNA expression and activation of TLR3 and TLR4 genes by RT-PCR. IL-8 production in the supernatants was determined by ELISA. Expression of IL-8 mRNA was significantly (*P< 0.05*) upregulated in HEK-293 cells expressing TLR4 when treated with *A. castellanii* trophozoites. *A. castellanii* did not upregulate TLR3 mRNA in TLR3 expressing HEK-293 cells (data not shown). IL-8 protein production was also increased significantly (*P< 0.05*) in TLR4 expressing cells after treatment with trophozoites (**Figure 4**). No major differences were seen in the TLR3 expressing HEK-293 in IL-8 mRNA or protein production when treated with *A. castellanii* as compared to untreated cells. IL-8 mRNA expression increased significantly (*P< 0.05*) in TLR3 and TLR4 expressing HEK-293 cells when treated with poly (I:C) and LPS, respectively, as compared to untreated cells. These results indicate that *A. castellanii* trophozoites activate TLR4 gene expression in TLR4 transfected HEK-293 cell lines resulting in enhanced IL-8 production.

### TLR4 Neutralizing Antibody Inhibits Proinflammatory Cytokine Production

**Figure 5:**
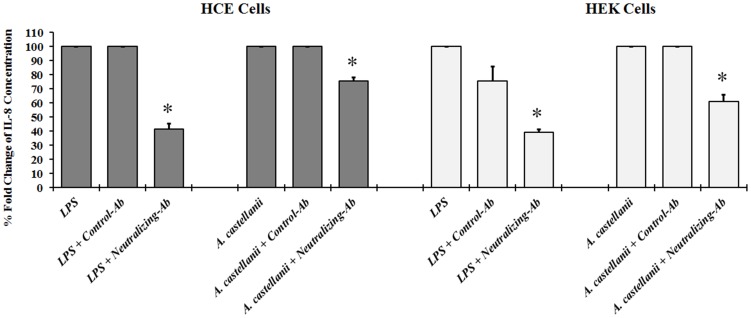
Effect of TLR4 neutralizing antibody on IL-8 production. HCE cells incubated with or without *A. castellanii* (1×10^5^ cells/ml) or LPS (10 μg/ml) for 24 hours. Inhibition of TLR4 activity was carry out by pre-incubating HCE cells for 1 hour with neutralizing TLR4 antibody (10 μg/ml) of anti-hTLR4 affinity purified goat IgG, or with the control antibody (10 μg/ml) normal human IgG, followed by incubation with or without *A. castellanii* (1×10^5^ cells/ml) or LPS (10 μg/ml) for 24 hours. HCE cells incubated without treatment, served as control untreated group. Supernatants were collected and IL-8 secretion was quantified by ELISA. Data are mean±SEM expressed in % fold change of IL-8 production of three independent experiments. *Asterisk* indicates *P* value < 0.05 by unpaired Student’s *t*-test.

To determine if TLR4 was responsible for the *Acanthamoeba* associated inflammatory response, HCE and TLR4 transfected HEK-293 cells were treated with a TLR4 neutralizing monoclonal antibody (anti-TLR4 mAb) and IL-8 production was quantified by ELISA. The anti-TLR4 mAb significantly blocked *Acanthamoeba* and LPS induced IL-8 production in both HCE and HEK-293 cells, while an isotype-matched control antibody was ineffective (**Figure 5**). Taken together, our findings reveal that TLR4 is involved in LPS and *Acanthamoeba* signaling in HCE and TLR4 transfected HEK-293 cells.

### Do Ocular But not Soil Isolates of *Acanthamoeba* Activate TLRs on Chinese Hamster Corneal Epithelial Cells and Induce Proinflammatory Cytokine CXCL2 Secretion?

**Figure 6:**
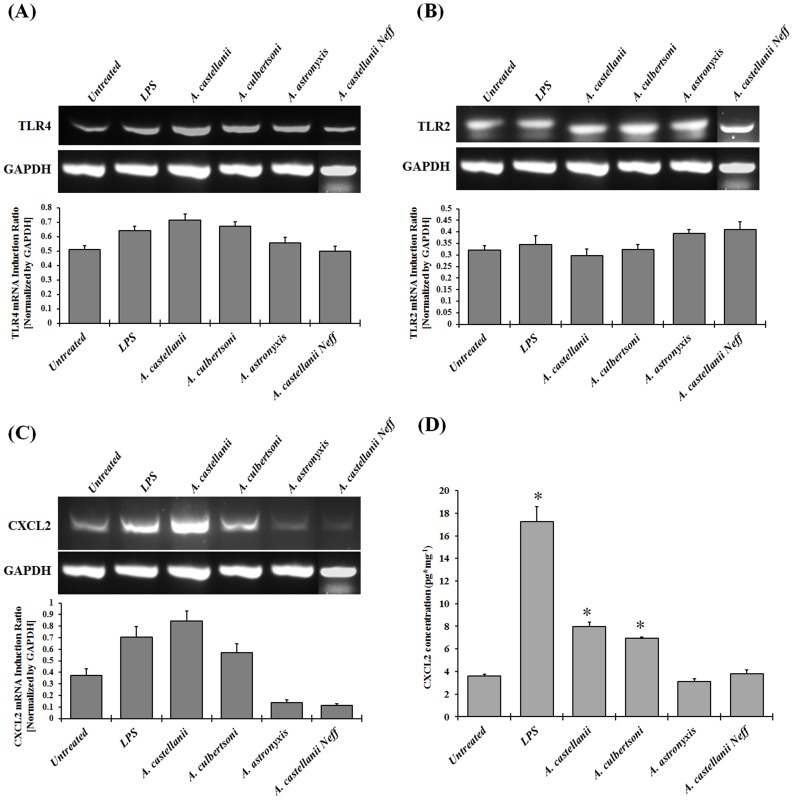
Pathogenic but not non-pathogenic isolates of *Acanthamoeba* upregulate TLR4 and CXCL2 mRNA, and induce CXCL2 secretion in Chinese hamster corneal epithelial (HCORN) cells. HCORN cells were stimulated with or without pathogenic (Clinical) isolates, *A. castellanii* (1×10^5^ cells/ml) and *A. culbertsoni* (1×10^5^ cells/ml), and non-pathogenic (Soil) isolates, *A. castellanii* Neff (1×10^5^ cells/ml) and *A. astronyxis* (1×10^5^ cells/ml), and LPS (10 μg/ml) for 24 hours incubation. Cells cultured without the trophozoites served as a control. Cells and supernatants were collected. Total RNA was isolated from cells and RT-PCR was performed to examine the mRNA expression of TLR2, TLR4, and CXCL2. Quantification was calculated by densitometry of bands relative to GAPDH. Densitometry of mRNA bands were quantified by three independent scanned presented as mean±SEM (6A – 6C). Supernatants from the harvested cells were subjected to CXCL2 specific ELISA (6D). The data are mean±SEM of three independent experiments. *Asterisk* indicates *P* value < 0.05 by unpaired Student’s *t*-test.

To determine whether *Acanthamoeba* trophozoites modulate the expression of TLR2, TLR4, and CXCL2 on Chinese hamster corneal epithelial (HCORN) cells, HCORN cells were stimulated with either pathogenic (Clinical isolate) *A. castellanii* and *A. culbertsoni*, or non-pathogenic (Soil isolate) *A. astronyxis* and *A. castellanii* Neff strains of *Acanthamoeba* trophozoites for 24 hours. The expression of TLR2, TLR4, and CXCL2 were then examined by RT-PCR. The secretion of CXCL2 production by cultured HCORN cells was examined by ELISA. Clinical isolates of *Acanthamoeba* but not soil isolates activate TLR4 and CXCL2 expression in the HCORN cells, while TLR2 expression was unchanged (**Figure 6A-6C**). Moreover, clinical isolates of *Acanthamoeba* (*A. castellanii* and *A. culbertsoni)* but not soil isolates (*A. astronyxis* and *A. castellanii* Neff) induced significant CXCL2 secretion from HCORN cells (**Figure 6D**). These results indicate that clinical isolates of *Acanthamoeba* but not soil isolates activate TLR4 expression and induce significant higher amount of CXCL2 secretion when treated with pathogenic strain of *Acanthamoebea* trophozoites.

### Upregulation of TLR4 and Proinflammatory Cytokine in the Chinese Hamster Model of *Acanthamoeba* Keratitis

**Figure 7:**
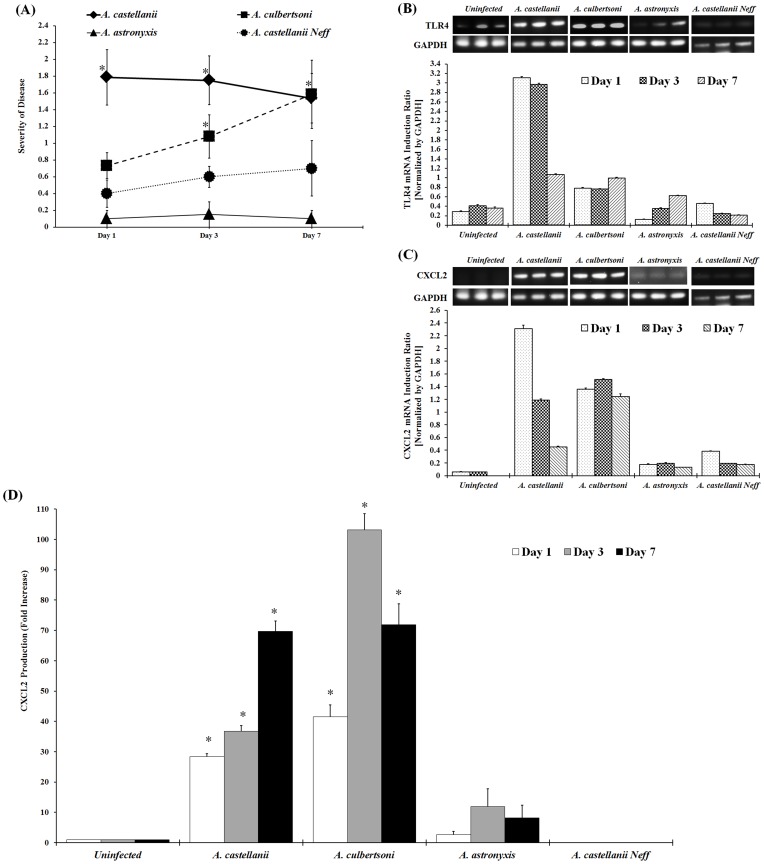
Pathogenic versus non-pathogenic recognition of TLR4 mediated proinflammatory cytokine CXCL2 production by *Acanthamoeba* in Chinese hamster. Chinese hamsters (n  =  6/group) were infected with either pathogenic isolate of *Acanthamoeba* trophozoites (*A. castellanii* or *A. culbertsoni*) or non-pathogenic isolate of *Acanthamoeba* trophozoites (*A. castellanii* Neff or *A. astronyxis*)-laden contact lenses as described earlier [7]. Lenses were removed 1, 3, and 7 days post-infection. (7A) Corneas were scored for severity of disease for the period indicated. Each graph line represents the mean±SEM of severity of disease of 6 animals for the observed time points. The results shown are representative of three separate experiments. *Asterisk* indicates *P* value < 0.05 by the Mann-Whitney’s *U* test. (7B and 7C) Infected and uninfected-control corneas were dissected and homogenized. Total RNA was collected and RT-PCR was performed to examine the mRNA expression of TLR4 and CXCL2. Quantification was calculated by densitometry of bands relative to GAPDH. Densitometry of mRNA bands were quantified by three independent scanned presented as mean±SEM. (7D) CXCL2 protein production from dissected and homogenized infected and uninfected-control corneal lysates was quantified using ELISA. The data are mean±SEM of three independent experiments. *Asterisk* indicates *P* value < 0.05 by unpaired Student’s *t*-test.

We have previously shown that *Acanthamoeba* soil isolates did not induce severe keratitis in Chinese hamsters and therefore, were categorized as nonpathogenic strains [2]. However, pathogenic strains of *Acanthamoeba* isolated from infected patients produced severe disease in Chinese hamsters, and thus were categorized as pathogenic strains. Moreover, infection with pathogenic strains of *Acanthamoeba* was associated with severe inflammation and infiltration of macrophages and neutrophils in the cornea. By contrast, inflammatory cells were absent in the corneas of animals infected with the soil isolates [2]. To determine whether TLR4 and chemokine CXCL2 play a role in infection process, Chinese hamsters (n  =  6) were infected with either pathogenic (*A. castellanii* or *A. culbertsoni*) or non-pathogenic (*A. astronyxis* or *A. castellanii* Neff) strains of *Acanthamoeba* as described previously [7]. Contact lenses were removed 1, 3, and 7 days postinfection, and corneas were scored for severity of disease for the period indicated. Each graph line represents the mean severity of the observed time points. Pathological observations showed that *A. castellanii* and *A. culbertsoni* infection but not *A. astronyxis* and *A. castellanii* Neff infection induced severe keratitis in Chinese hamsters (**Figure 7A**).

Corneas from each group were dissected at the indicated time after infection and the expression of TLR4 and CXCL2 were analyzed by RT-PCR. *A. castellanii* and *A. culbertsoni* but not *A. castellanii* Neff and *A. astronyxis* induced significant upregulation of TLR4 and CXCL2 in the cornea of Chinese hamsters on day 1, 3, and 7 postinfection (**Figure 7B and 7C** **)**. Chinese hamster corneas infected with either pathogenic or non-pathogenic strains of *Acanthamoeba* trophozoites were examined for CXCL2 secretion. *A. castellanii* and *A. culbertsoni* but not *A. castellanii* Neff and *A. astronyxis* trophozoites induced significant CXCL2 production in Chinese hamster corneas 1, 3, and 7 days after infection (**Figure 7D**). These results suggest that clinical isolates of *Acanthamoeba* species but not soil isolates recognize TLR4 and induce proinflammatory cytokine production during *Acanthamoeba* keratitis (AK).

### Histological Evaluation of *A. castellanii* Induced Pathological Process

**Figure 8:**
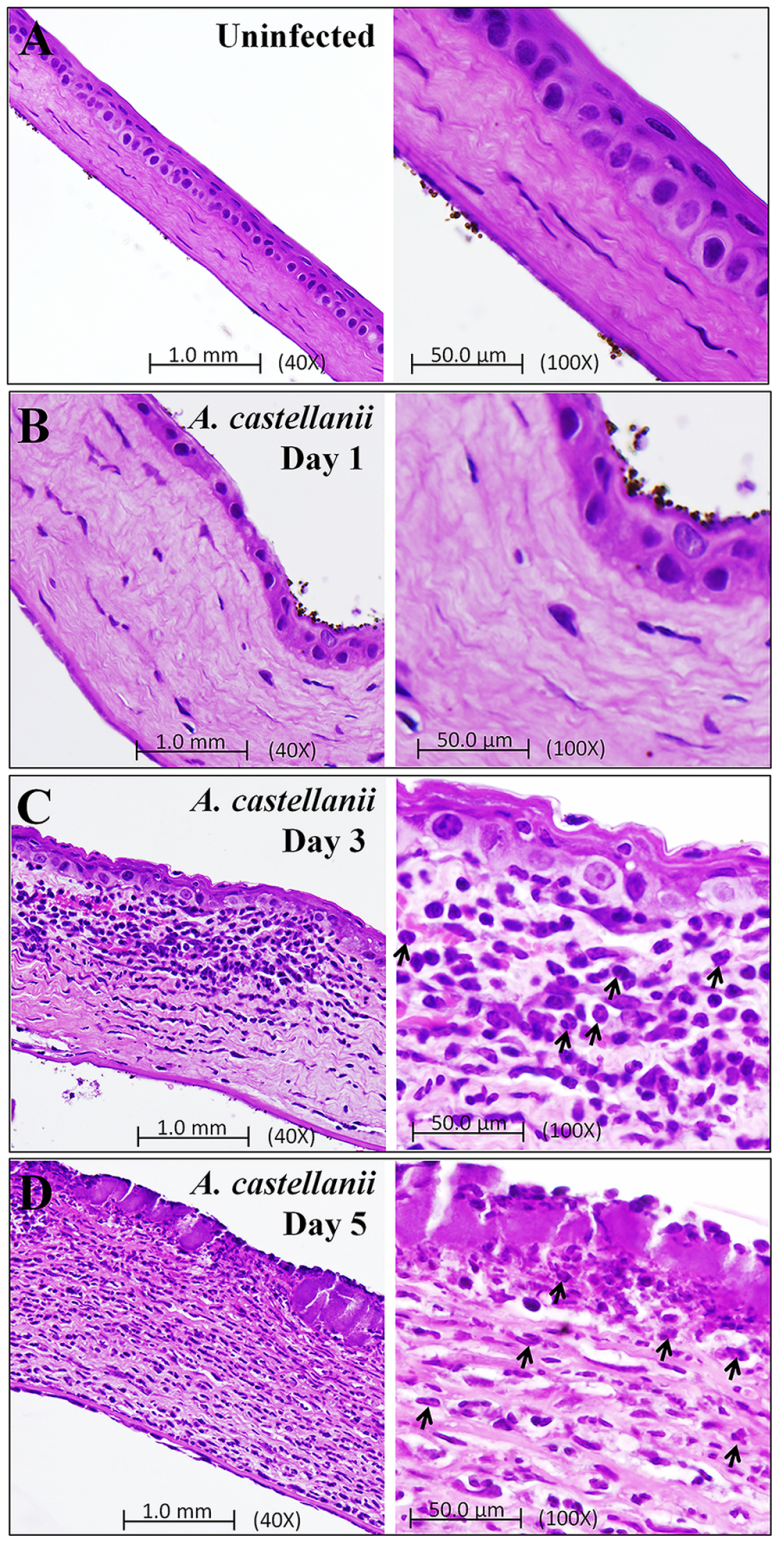
Pathological evaluation of *A. castellanii* induced inflammation in Chinese hamster corneas. Photomicrograph of corneas from a Chinese hamster infected with *A. castellanii* trophozoites-laden contact lenses. Animals were anesthetized and sacrificed on 1, 3, and 5 days postinfection. The histopathological features of the uninfected and infected corneas included - (**8A**) Cornea from control-uninfected animal was normal with regular pattern of corneal epithelium, stroma, and corneal endothelium. (**8B**) Infected corneal section of day 1 postinfection shows very mild inflammation in stroma as compared to uninfected corneal section. (**8C** and 8**D**) Infected corneal section of 3 and 5 days postinfection show epithelial ulceration, focal thickening, PMNs infiltration, corneal thickening, marked lamellar connective tissue disruption, and extensive stroma swelling when compared with uninfected corneal section. PMN cells (Arrows) were present in the stroma. (**8A-8D**) Left panel shows 40X magnification and right panel shows 100X magnification.

Infected and uninfected corneas were collected from Chinese hamsters on day 1, 3, and 5 postinfection for corneal histopathologic analysis. The histopathologic features of the infected corneas 3 and 5 days postinfection showed that epithelium and stroma were markedly infiltrated by a large number of inflammatory cells and the structure of corneal epithelium became unorganized as compared with uninfected corneas. The corneal stroma in infected corneas contained marked lamellar connective tissue disruption and thickening 3 and 5 days after infection. Severe PMN cells infiltration were evident on day 3 postinfection. By contrast, corneal epithelium was normal and milled stroma swelling was observed in the corneal stroma on day 1 postinfection (**Figure 8**). Histopathlogic examination of corneas from animal infected with non-pathogenic strains of *Acanthamoeba* (*A. astronyxis* and *A. castellanii Neff*) revealed no significant inflammatory cells infiltration in the corneas (data not shown).

## Discussion

The host’s immune response begins with the recognition of a foreign pathogen. After recognition, a signaling cascade is activated that produces proinflammatory cytokines and chemokines to combat the infection. Several groups have shown that TLRs are the PRRs that recognize pathogens during ocular infection [17], [18]. In this study, we aimed to determine if TLRs are responsible for the recognition of *Acanthamoeba* trophozoites during AK. We also investigated whether non-pathogenic stains of *Acanthamoeba* are recognized by TLRs in the same manner. The present study is the first to compare the *in vitro* and *in vivo* activation of TLR4 simultaneously in response to pathogenic and non-pathogenic *Acanthamoeba* infection.

Our results showed that TLR4 is upregulated on HCE cells when treated with *A. castellanii* trophozoites for 24 hours. *Acanthamoeba* species are extracellular pathogens that attach to corneal epithelial cell surfaces. The amoebae do not become intracellular and do not have flagellum, which decreases the possibility for the parasite to be recognized by TLR3 and TLR5, which are activated by double-stranded RNA and bacterial flagellin, respectively, and are also present and function in human corneal epithelial cells [22], [29]–[32]. Our studies indicated that interaction of *Acanthamoeba* with the corneal epithelial cells induces a rapid immune response by the production of IL-8, which can initiate efficient host inflammatory responses to corneal infections.

Immunostaining of HCE cells revealed that TLR4 is significantly upregulated 24 hours after *Acanthamoeba* challenge. Pretreatment of HCE and TLR4 transfected HEK-293 cells with TLR4 neutralizing antibody mitigated the increased IL-8 production after stimulation with *Acanthamoeba* trophozoites. The use of a TLR4 neutralizing antibody did not bring the IL-8 production level down to a basal level in HCE cells. These findings indicated that either other TLRs are involved in the recognition of *Acanthamoeba* and induction of IL-8 or there is an insufficient antibody to block TLR4. Given that, TLR4 antibody did not completely block IL-8 production in TLR4 transfected HEK-293 cell line; we believe the later to be rationale for our observation. These findings are in agreement with those of Ren *et al* [33] who found TLR4 was the main receptor that upregulated in human corneal epithelial cells and induced upregulation of IL-8 and other cytokines after *Acanthamoeba* challenge. Our results not only confirmed their findings but also demonstrated that pathogenic strains of *Acanthamoeba* are recognized by TLR4 in Chinese hamster model of *Acanthamoeba* keratitis. To confirm that TLR4 are involved in the recognition of *Acanthamoeba* and mediate signaling responses to *A. castellanii* trophozoites, TLR3 and TLR4 transfected HEK-293 cells were exposed to *A. castellanii in vitro* for 24 hours. Expression of IL-8 mRNA was significantly upregulated in HEK-293 cells expressing TLR4 when treated with *A. castellanii* trophozoites. No significant differences were seen in the TLR3 expressing HEK-293 in IL-8 mRNA or protein production. IL-8 protein production also increased significantly in TLR4 expressing cells after treatment with *A. castellanii* trophozoites. The underlying mechanisms that regulate corneal epithelial cell activation are therefore important in the development of keratitis.

Song *et al* [24] demonstrated that TLR4 is localized on the cell surface of human corneal epithelial cells and that LPS induces production of proinflammatory and chemotactic cytokines. In contrast, Ueta *et al* [34] reported that TLR2 and TLR4 are intracellular, and demonstrated that LPS and peptidoglycan do not stimulate cytokine production above background levels. Although the discrepancy between these studies is yet to be resolved, the results of the present study clearly indicated that activation of TLR4 on corneal epithelial cells *in vitro* and *in vivo* stimulate cytokine production and development of keratitis.

Interestingly, both *in vivo* and *in vitro* results demonstrated that soil isolate of *Acanthamoeba* (*A. astronyxis* and *A. castellanii* Neff strain) but not clinical isolate (*A. castellanii* and *A. culbertsoni*) failed to the upregulation of TLR2, TLR4, and CXCL2 gene expression. However, *A. castellanii* and *A. culbertsoni* trophozoites induced significant CXCL2 protein production in Chinese hamster corneas 1, 3, and 7 days postinfection. Since mutation in TLR2 makes TLR2 is non-functional in Chinese hamsters [35], [36]. These results suggest that TLR4 is responsible for pathogen recognition in Chinese hamster model of *Acanthamoeba* keratitis.

TLR family members are transmembrane proteins containing repeated leucine-rich motifs in their extracellular portions, similar to other pattern recognition proteins of the innate immune system [37], [38]. TLRs also contain a cytoplasmic domain, which is homologous to the signaling domain of IL-1 receptors, and activation of TLRs result in activation of NF-kB and induction of cytokines and co-stimulatory molecules for the activation of the adoptive immune response [37]–[39]. Our data do not preclude a contributory role for other TLR family members but agree with genetic evidence in hamsters, in which a mutation in TLR2 makes TLR2 non-functional in Chinese hamsters [35], [36]. Although we cannot eliminate the possibility that TLR2 might contribute to the pathogenicity of AK, it is clear that TLR4 is the dominant receptor.

We predicted that the initial role of TLR4 in AK is to activate corneal epithelial cells that produce chemotactic and proinflammatory cytokines which mediate neutrophil recruitment to the corneal stroma. It has been shown that neutrophils express most TLRs [40], a second role for this pathway would therefore be *Acanthamoeba*-induced activation of neutrophils and further production of CXCL2 chemokines in the corneal stroma. Activated neutrophils would also degranulate and release cytotoxic mediators, such as nitric oxide and myeloperoxidase, which cause tissue damage and loss of corneal clarity. IL-8 and CXCL2 are chemoattractants known to attract PMN to a site of infection. Although the current study has focused on the role of proinflammatory chemokines in AK, it is clear that other pathways are also involved. For example *P. aeruginosa* induced expression of antimicrobial peptides, including β-defensins, in corneal epithelial cells *in vivo* [41], [42]. Furthermore, pre-treatment of corneas with isolated flagellin inhibits *P. aeruginosa* keratitis by stimulating production of antimicrobial peptides, such as cathelicidin [43], [44]. It has been shown that mice deficient in macrophage migratory inhibition factor failed to produce cytokine production and neutrophil infiltration to the cornea after *P. aeruginosa* infection [45].

Histological features of *Acanthamoeba* infected corneas from 1, 3, and 5 days showed mild to consistently severe infiltration of inflammatory cells (PMN) which induces lamellar connective tissue disruption and increases thickening of corneal stroma, and further leads to disruption of corneal epithelium. Thus, outcomes of PMN infiltration in corneas strongly support increased level of CXCL2 in Chinese hamster corneas 3 and 7 days after *Acanthamoeba* infection. During AK, PMN recruitment is critical to disease severity. In several models of corneal inflammation, the roles of neutrophils appear as a double-edged sword, with one edge fighting the invading pathogens and the other triggering tissue damage of the host. Because the mammalian TLR family has many members, it is possible that the innate immune system and its individual cell types use different combinations of TLRs to recognize different groups of microbial pathogens. Finally, our findings indicated that TLR4 may be a potential target in the development of novel treatment strategies in *Acanthamoeba* and other microbial infection that activate TLR4 in corneal epithelial cells.

## Materials and Methods

### Ethics statement

Animals were handled in accordance with the Association of Research in Vision and Ophthalmology “Statement on the Use of Animals in Ophthalmic and Vision Research” (http://www.arvo.org/About_ARVO/Policies/). All animal experiments were conducted and handled in strict accordance with good animal practice as defined by the relevant national and/or local animal welfare bodies, and all animal studies were approved by University of North Texas Health Science Center Institutional Animal Care and Use Committee (IACUC). The ethics approval (Protocol/permit/project license number “2010/11-23-A06) for the animal experiments was granted on February 19, 2013.

### Animals

Chinese hamsters were purchased from Cytogen Research and Development Inc., West Roxbury, MA, USA. All animals used were from 4 to 6 weeks of age and all corneas were examined before experimentation to exclude animals with preexisting corneal defects. All procedures were performed on the left eyes. The right eyes were not manipulated.

### Amoebae and Cell Lines

Clinical isolates of *Acanthamoeba* species, *Acanthamoeba castellanii* (ATCC 30868) and *Acanthamoeba culbertsoni* (ATTC 30171), and soil isolates, *Acanthamoeba castellanii* Neff (ATCC 30010) and *Acanthamoeba astronyxis* (ATTC 30137), were obtained from the American Type Culture Collection (ATCC), Manassas, VA, USA. Amoebae were grown as axenic cultures in peptone-yeast extract-glucose at 35°C with constant agitation on a shaker incubator at 125 rpm [46]. Human telomerase-immortalized corneal epithelial (HCE) cells [47], a gift from James Jester, Ph.D. (University of California, Irvine), were cultured in keratinocyte medium (KBM-2 Bullet Kit; BioWhittaker, Lonza, Walkersville, MD, USA) containing 10% fetal bovine serum (Hyclone, Logan, UT), at 37°C with 5% CO_2_. Chinese hamster corneal epithelial (HCORN) cells were immortalized with human papillomavirus E6 and E7 genes, as previously described [48], [49] and cultured in complete minimum essential medium (MEM; BioWhittaker, Lonza, Walkersville, MD, USA) containing 1% L-glutamine, 1% penicillin, streptomycin, amphotericin B, 1% sodium pyruvate (BioWhittaker, Lonza, Walkersville, MD, USA), and 10% fetal calf serum (FCS, HyClone Laboratories, Inc., Logan, UT), at 37°C in a humidified 5% CO_2_. Human embryonic kidney 293 (HEK-293) cells stably transfected with TLR3 or TLR4 were obtained from Eicke Latz, Ph.D (University of Massachusetts Medical School, Worchester, MA) and maintained in Dulbecco’s modified Eagle’s medium (DMEM) containing 4.5 g/liter glucose with L-glutamine (BioWhittaker, Lonza, Walkersville, MD, USA), 10% fetal calf serum (FCS, HyClone Laboratories, Inc., Logan, UT) and 10 μg/ml Cipro (Cellgro Mediatech, Inc., Manassas, VA) at 37°C with 5% CO_2_ [28].

### Cell Cultures and Treatment Experiments

HCE cells were cultured in 24 well plates until confluent. Once confluent, cells were stimulated with or without *A. castellanii* (1×10^5^ cells/ml) and Ultra-pure LPS (10 μg/ml) (InvivoGen, San Diego, CA, USA) for 24 hours. HEK-293 cells were exposed to *A. castellanii in vitro*. *A. castellanii* trophozoites (1×10^5^/ml) were added to 24 well plates containing HEK-293 cells (1×10^6^ cells/ml) for 24 hours. Cells cultured without the trophozoites served as a control. As a positive control, transfected HEK-293 cells were activated with 125 ng/ml poly (I:C) (polyinosinic:polycytidylic acid), a specific ligand for TLR3 (InvivoGen, San Diego, CA, USA); and 100 ng/ml ultrapure LPS, a specific ligand for TLR4/MD2 (InvivoGen, San Diego, CA, USA) for 24 hours as described previously [28]. HCORN cells were cultured in 24 well plates until confluent and then cells were stimulated with or without *A. castellanii* (1×10^5^ cells/ml), *A. culbertsoni* (1×10^5^ cells/ml), *A. astronyxis* (1×10^5^ cells/ml), *A. castellanii* Neff (1×10^5^ cells/ml) and Ultra-pure LPS (10 μg/ml) (InvivoGen, San Diego, CA, USA) for 24 hours.

Cells and supernatants were collected by centrifugation at 2000×g for 10 min at 4°C and analyzed for the expression of TLR1, TLR2, TLR3, TLR4, TLR9, IL-8, CXCL2 and GAPDH genes by RT-PCR. IL-8 and CXCL2 production in the supernatants was determined by ELISA.

### *In vivo* Infection

*Acanthamoeba* keratitis (AK) was induced in Chinese hamster corneas as previously described [7]. Briefly, Chinese hamsters were anesthetized with ketamine (115 mg/kg) and xylazine (10 mg/kg) (Fort Dodge Laboratories, Fort Dode, Iowa, USA) injected intraperitoneally (IP). A topical anesthetic, Alcain (Alcon Laboratories, Fort Worth, Texas, USA) was used to anesthetize the corneas prior to being abraded with a sterile cotton applicator. Contact lenses laden with either clinical isolates (*A. castellanii* or *A. culbertsoni*) or soil isolates (*A. astronyxis* or *A. castellanii* Neff) were placed onto the center of the cornea and the eyelids were closed by tarsorrhaphy with 6-0 Ethilon sutures (Ethicon, Somerville, New Jersey, USA). Lenses were removed 1, 3, and 7 days postinfection, and corneas were scored for severity of disease. The pathology was scored on a scale of 0 – 5 based on the following parameters: corneal infiltration, corneal defect, corneal neovascularization, and corneal ulceration. The pathology was recorded as: 0  =  no pathology, 1  =  <10% of the cornea involved, 2  =  10 – 25% involved, 3  =  25 – 50% involved, 4  =  50 – 75% involved, and 5  =  75 – 100% involved, as described previously [7]. Any animals receiving a score of at least 1.0 for any parameter were scored as infected. Corneas were removed from infected Chinese hamsters on day 1, 3, and 7 post-infection (n  =  6 for each time point) after recording the pathology. All pieces of the limbus were removed and tissues were disrupted by sonication (Sonicator Model CL-18, Fisher Scientific, Pittsburgh, PA, USA) as described previously [6]. Corneal pellets and corneal lysates were collected by centrifugation at 2000×g for 10 minutes at 4°C, and analyzed for the expression of TLR4, CXCL2, and GAPDH genes by RT-PCR. CXCL2 production in the cell lysates was determined by ELISA.

### Isolation of RNA and Reverse Transcription-PCR

Total RNA was isolated from cells and corneal pellets using Trizol reagent (Invitrogen, Carlsbad, USA) as described previously [50], [51]. cDNA was synthesized using 2 μg of total RNA using a High Capacity cDNA Reverse Transcription kit (Applied Biosystems, Carlsbad, CA). PCR was performed using AmpliTaq Gold PCR Master Mix (Applied Biosystems, Carlsbad, CA). Toll-like receptor genes were amplified using the following cycling conditions: initial denaturation at 94°C for 5 minutes, 40 cycles of denaturation at 94°C for 15 seconds, primer annealing at 60°C for 15 seconds, and extension at 72°C for 15 seconds, and a final extension at 72°C for 5 minutes. Cycling conditions for cytokines were 94°C for 5 minutes, followed by 40 cycles of denaturation at 94°C for 1 minute, primer annealing at 60°C for 1 minute, and extension at 72°C for 1 minute, and one final extension at 72°C for 10 minutes. All mRNA expression was normalized to GAPDH as an internal control. Gene expression was quantified by Vision Works LS Image Acquisition & Analysis software (UVP, LLC, Upland, CA) and the results expressed as a fold increase over GAPDH gene expression. The human oligonucleotide primers used are as follows: TLR1 (615 bp): 5′-ACGGTCTCATCCACGTTCCTAAAGA-3′ (sense), 5′-CGCCAGAATACTTAGGAAGTAAGAAC-3′ (anti-sense); TLR2 (380 bp): 5′-TGGAGAGACGCCAGCTCTGGCTCA-3′ (sense), 5′-CAGCTTAAAGGGCGGGTCAGAG-3′ (anti-sense); TLR3 (304 bp): 5′-GATCTGTCTCATAATGGCTTG-3′ (sense), 5′-GACAGATTCCGAATGCTTGTG-3′ (anti-sense); TLR4 (142 bp): 5′-GATTGCTCAGACCTGGCAGTT-3′ (sense), 5′-TGTCCTCCCACTCCAGGTAAGT-3′ (anti-sense); TLR9 (260 bp): 5′-GTGCCCCACTTCTCCATG-3′ (sense), 5′-GGCACAGTCATGATGTTGTTG-3′ (anti-sense); IL-8 (289 bp): 5′-ATGACTTCCAAGCTGGCCGTGGCT-3′ (sense); 5′-TCTCAGCCCTCTTCAAAAACTTCTC-3′ (anti-sense); GAPDH (450 bp): 5′-ACCACAGTCCATGCCATCAC-3′ (sense); 5′-TCCACCACCCTGTTGCTGTA-3′ (anti-sense). Mouse oligonucleotide primers used are CXCL2 (285 bp) 5′-ACCCTGCCAAGGGTTGACTTC-3′ (sense), 5′-GGCACATCAGGTACGATCCAG-3′ (anti-sense) as described earlier [50]. The Chinese hamster oligonucleotide primers used are as follows: TLR2 (413 bp, Accession number XR_135860.1): 5′-TCTCTCCAGGAAGGGATGTTCTG-3′ (sense), 5′-GGGTCTGTAAATGTGTGAGGTTGAG-3′ (anti-sense); TLR4 (201 bp, Accession number NM_001246762.1): 5′-TGCTCAGACATGGCAGTTTC-3′ (sense), 5′-GCTCTTCCATCCAACAGAGC-3′ (anti-sense); GAPDH (333 bp): 5′-CAAGTTCAAAGGCACAGTCAA-3′ (sense), 5′-GTGAAGACGCCAGTAGATTCC-3′ (anti-sense) Chinese hamster GAPDH (333 bp) available in the UniProtKB/Swiss-Prot database (http://www.uniprot.org/uniprot/P17244). All primers were verified by BLAST (Basic Local Alignment Search Tool, in the public domain, http://blast.ncbi.nlm.nih.gov/Blast.cgi). All primers were from Integrated DNA Technologies, Inc. (Commercial Park, Coralville, Iowa, USA).

### Enzyme-Linked Immunosorbent Assay (ELISA)

IL-8 and CXCL2 cytokine production was quantified from cell supernatants and corneal lysates using an ELISA kit (R&D System, Minneapolis, MN) as described previously [50], [51]. Briefly, cell culture supernatants and cell lysates were collected at the indicated periods after treatments and centrifuged to remove cell debris. Total protein concentrations of each supernatant and lysate were determined by bicinchoninic acid assay (BCA assay) [52]. The absorbance was measured at 450 nm using an ELISA reader (Gen5 1.10, BioTek Instruments Inc., Winooski, Vermont, USA). The minimum detectable dose of CXCL2 by ELISA was <1.5 pg/ml and the minimum detectable dose of IL-8 by ELISA was 1.5-7.5 pg/ml. The results were expressed in pg/mg of protein.

### TLR4 Neutralizing Antibody Treatment

HCE and HEK-293 cells were cultured in 24 well plates at approximate 90% confluence in an appropriate medium and incubated with or without *A. castellanii* (5×10^5^ cells/ml) or LPS (10 μg/ml) for 24 hours. Inhibition of TLR4 activity was carried out by pre-incubating HCE cells for 1 hour with neutralizing TLR4 antibody (10 μg/ml of anti-hTLR4 affinity purified goat IgG (R&D Systems, Minneapolis, MN) or with the control antibody (10 μg/ml of normal human IgG Control (R&D Systems, Minneapolis, MN) followed by incubation with or without *A. castellanii* (5×10^5^ cells/ml) or LPS (10 μg/ml) for 24 hours. HCE cells incubated without treatment, served as the control untreated group. Supernatants were collected by centrifugation and the level of IL-8 was measured by ELISA. Results were expressed in % Fold change of IL-8 concentration.

### Immunocytochemistry Method

HCE or HEK-293 cells were grown to confluence on 4 well chamber slides (Thermo fisher, Rochester, NY). HCE cells were stimulated with 1×10^5^/ml of *A. castellanii* trophozoites for 24 hours. HCE control cells and HEK-293 (positive control) cells were left untreated for 24 hours at 37°C. After the incubation period, cells were fixed with 4% paraformaldehyde, washed with PBS, blocked with 5% fetal bovine serum in phosphate buffered saline (PBS), washed and then incubated with either PE anti-human TLR4 or PE Mouse IgG2α isotype control (BioLegend, San Diego, CA, USA) for 1 hour. To observe results, the nuclei sections were counterstained for 1 minute in 150 ng 4,6-diamidino-2-phenylindole, dilactate (DAPI, Calbiochem, Darmstadt, Germany) and washed with PBS. Three slides in each group were viewed using fluorescence microscopy. Images were captured with an Olympus AX70 Fluorescence Microscope. The results were expressed as a percentage of cells positive for TLR4 by counting the number of TLR4 positive cells divided by the number of live cells multiplied by 100. Cells stained with DAPI were counted as live cells.

### Histological Examination of *A. castellanii* Infected Cornea

Chinese hamsters (n  =  6/group) were infected with *A. castellanii* trophozoites-laden contact lenses as previously described [7]. Animals were anesthetized and sacrificed 1, 3, and 5 days after infection. Each animal right eye (uninfected) was used as control. Eyes were removed and stored in 10% Carson’s formalin for 24 hours. Specimens were then embedded in paraffin, cut into 4 mm sections using a Reichert Histostat rotary microtome (Reichert Scientific Instruments, Buffalo, NY), and placed on polysine hydrobromide-precoated slides (Polysciences,Warrington, Pa.). Sections were stained with hematoxylin and eosin, covered with a coverslip, and examined by light microscopy. Pictures were taken by camera-enhanced light microscopy (BX50; Olympus Optical, Tokyo, Japan).

### Statistics

All experiments were performed in triplicate and results are presented as mean ± SEM. Differences between two groups were determined by unpaired Student’s *t*-test. While severity of disease scores were analyzed by the Mann-Whitney’s *U* test. In all analysis, *P* < 0.05 was considered as statistically significant. In all analysis, *P*< 0.05 was considered statistically significant.
